# ENGAGING RURAL VETERANS’ PERCEPTIONS OF SURVIVORSHIP CARE FOR HEAD AND NECK CANCER

**DOI:** 10.1093/geroni/igz052

**Published:** 2019-12-09

**Authors:** Aaron T Seaman, Julia Friberg, Jenna L Adamowicz, Nicholas Kendell, Nitin A Pagedar, M Bryant Howren, Alan J Christensen

**Affiliations:** 1 Iowa City VA Healthcare System, Iowa City, Iowa; 2 Center for Comprehensive Access and Delivery Research and Evaluation, Iowa City, Iowa; 3 University of Iowa, Iowa City, Iowa; 4 Rural Health Resource Center, Iowa City, Iowa

## Abstract

The purpose of this study was to investigate 1) rural patients’ perceptions of their own rurality and its effects on experience of head and neck cancer survivorship, and 2) potential barriers and facilitators to survivorship care within an integrated health care delivery system of the Veterans Health Administration (VHA). Data from qualitative interviews with Veterans who have a history of head and neck cancer are presented to understand the complex ways that rurality impacts cancer survivorship. Head and neck cancer survivors must contend with specific challenges resulting from their risk factors and treatment, including access to complex medical follow up, long-term physical and psychological effects of treatment, and tobacco- and alcohol-related comorbidities. While integration within the VHA facilitates coordination of specialty and primary care and the transfer of medical information, the use of community care in rural areas presents coordination challenges, especially for survivors with comorbidities.

